# Solitary fibrous tumor of male breast: A case report and literature review

**DOI:** 10.1097/MD.0000000000032199

**Published:** 2022-12-16

**Authors:** Shun Kawaguchi, Keiichi Kinowaki, Nobuko Tamura, Aya Nishikawa, Akio Shibata, Kiyo Tanaka, Yoko Kobayashi, Takuya Ogura, Junichiro Sato, Hidetaka Kawabata

**Affiliations:** a Department of Breast and Endocrine Surgery, Toranomon Hospital, Tokyo, Japan; b Department of Pathology, Toranomon Hospital, Tokyo, Japan.

**Keywords:** aged, immunohistochemistry, male, solitary fibrous tumors

## Abstract

Solitary fibrous tumors (SFT) are uncommon mesenchymal neoplasms which can arise in any anatomical location. Pleural SFTs have been most frequently documented; however, breast SFT is an exceedingly rare entity and seldom present in male breast, with only 8 previously reported cases. Recently, STAT6 immunostaining was considered to be a definitive marker of SFT, however, no case of the male breast SFT showing STAT6 positivity preoperatively has been reported.

**Methods and Results:**

We describe a case of breast SFT in a 73-year-old male patient with a 12-month history of a palpable breast mass. The only associated clinical symptom was bilateral gynecomastia. An ultrasound scan examination revealed an oval, well-circumscribed and hypoechoic mass with hypervascularity. A core-needle biopsy was performed, and microscopic examination with immunohistochemistry confirmed the diagnosis of SFT. He underwent a complete surgical resection with clear margins, and there were no signs of high cellularity, remarkable mitotic activity, pleomorphism, hemorrhage or necrosis.

**Conclusion:**

A perioperative immunohistochemical evaluation for diffuse and intense nuclear expression of STAT6 was helpful to distinguish SFT from myofibroblastoma. We, herein, describe the first case of SFT in a male breast, confirmed by STAT6 immunostaining positivity. We also conducted a literature review of all previous cases of breast SFTs.

## 1. Introduction

Solitary fibrous tumor (SFT) is an uncommon, mesenchymal tumor, most frequently detected in the pleural cavity and lungs. Breast SFT is an extremely rare entity, with only 32 cases previously reported in the English literature (see Table S1, http://links.lww.com/MD/I75, which summarizes mammary SFTs reported in the English literature). The histology of SFT is characterized by the pathologic feature of spindle cells showing pattern-less growth with rich stromal collagen and hemangiopericytic-like vascular branches; however, a definitive diagnosis of SFT based solely on histologic findings cannot be made since nonclassical SFT are frequently seen, showing atypical growth pattern with various stromal change. Immunohistochemistry (IHC) also showed overlapping expression patterns with other types of spindle cell tumor, such as myofibroblastoma and spindle cell lipoma.^[1,2]^

Recently, recurrent NAB2-STAT6 gene fusion, located at chromosomal region 12q13, was identified as a driver mutation of SFT,^[3,4]^ and Barthelmeß et al^[5]^ reported that a nuclear positivity for STAT6 was strongly associated with the presence of NAB2-STAT6 fusion gene, with a frequency of 92%, in a study of 52 patients with SFT. Diffuse and intense STAT6 nuclear staining may accurately distinguish breast SFT from other types of mesenchymal tumor; however, the utilization of STAT6 immunostain for the preoperative diagnosis of breast SFT has been reported in only 4 cases. Breast SFTs more commonly appeared in female than male patients, while we observed pleural SFT as common in male as in female patients. There remains a lack of evidence concerning the clinical courses and the prognosis of breast SFTs due to their rarity.

Here, we report the first case of male breast SFT, which was preoperatively identified by IHC of STAT6, with a review of literature. We described the diagnostic evaluation and the management of this exceedingly rare SFT with a series of imaging and pathologic findings. The patient has provided informed consent for publication of the case, and written informed consent for publication of the details was obtained from the patient and the next of kin.

## 2. Case presentation

**Figure 1.**
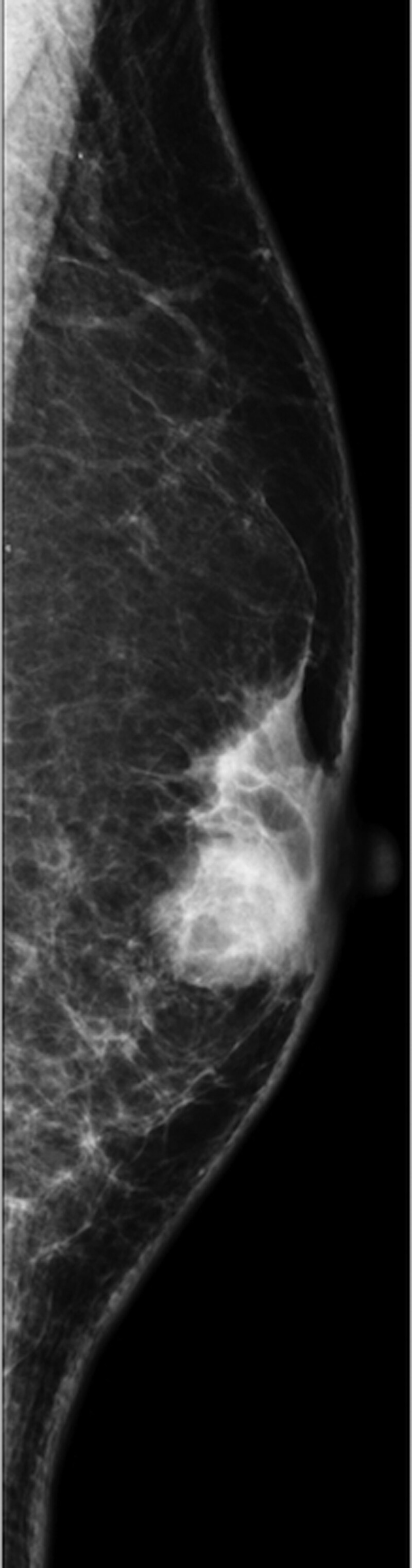
Mammogram showing an oval and well-circumscribed mass in the left breast at subareolar area.

The patient was a 72-years-old male with a 2.0 cm palpable breast mass, that increased in size over 12 months. The mass, localized to the subareolar area of the left breast, was not fixed to the thoracic wall, and showed a mild tenderness on palpation. Axillary lymph nodes were not palpable, and no skin modification or nipple discharge was observed. His medical and family history were uneventful, and he took no medications or nutritional supplements. He was 170 cm tall and weighed 62 kg. Laboratory data revealed normal liver and kidney functions, with serum tumor marker levels within normal range. Mammography showed a high-density round mass with well-defined borders (Fig. 1) and gynecomastia.

**Figure 2.**
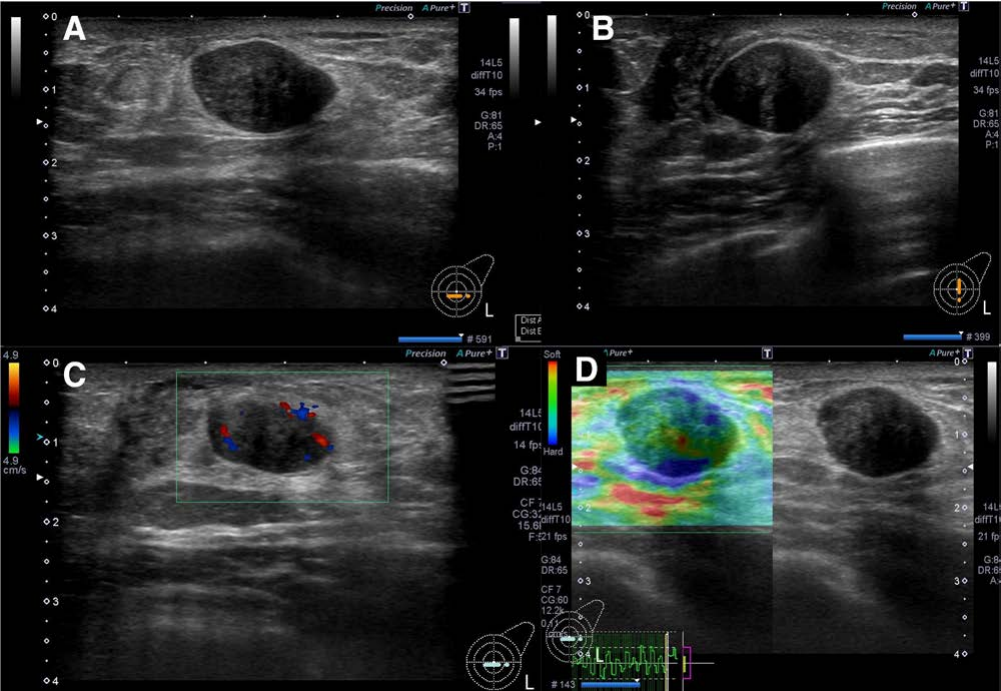
Breast ultrasound showing (a, b) an oval, well-circumscribed and hypoechoic mass measuring 20.8 × 17.8 × 12.4 mm with an anteroposterior/longitudinal ratio of 0.60. (c) Color doppler ultrasound reveals some central and peripheral color flow signals. (d) The strain elastography shows an overall intermediate hardness colored in green, consisting of heterogeneous hard and soft zone colored in blue or red.

**Figure 3.**
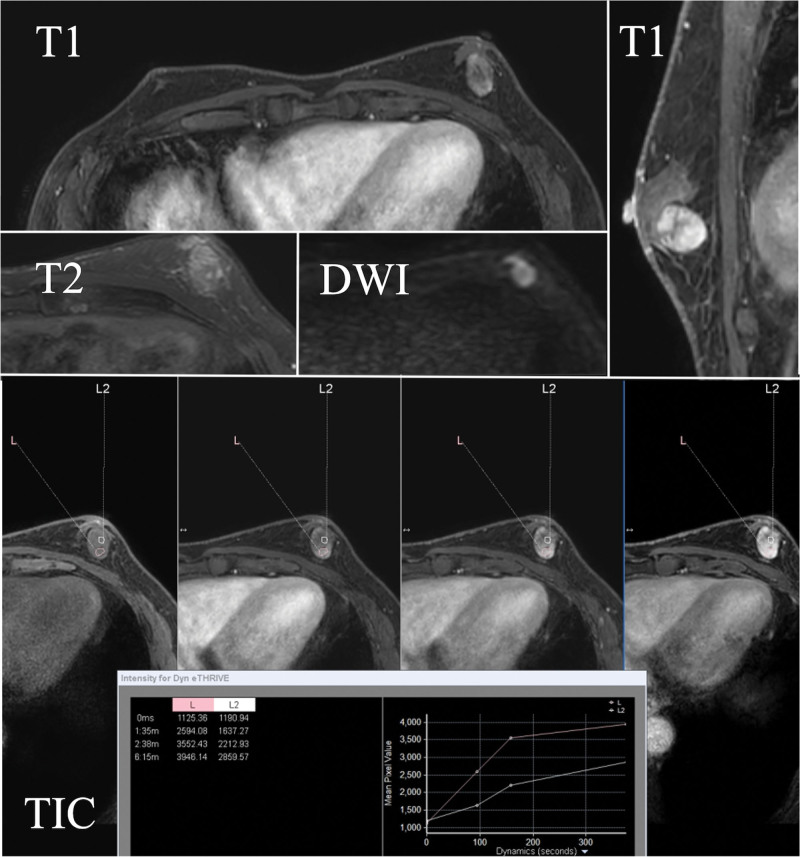
Magnetic resonance imaging: A well-defined and oval lesion was heterogeneously high signal intensity on T1WI and T2WI with time intensity curve of rapid-plateau pattern.

Ultrasonography revealed a 20.8 × 17.8 × 12.4 mm, oval and well-circumscribed, hypoechoic mass with central and peripheral blood flow (Fig. 2). Magnetic resonance imaging showed a well-defined, oval lesion with geographic enhancement (Fig. 3). A diagnosis of SFT was confirmed from the core-needle biopsy due to the intense and diffuse STAT6 nuclear positivity, and total mastectomy was indicated. We performed total mastectomy including the excision of the needle tract to avoid local recurrence due to iatrogenic implantation. Operation time was 38 minutes, and there was no remarkable blood loss. He was discharged on post-operative day 2, and there was no perioperative complication. The surgical margins were tumor free, and he was followed up every 6 months with no further treatment. He had no evidence of recurrence at 6 months postoperatively.

## 3. Pathological findings

**Figure 4.**
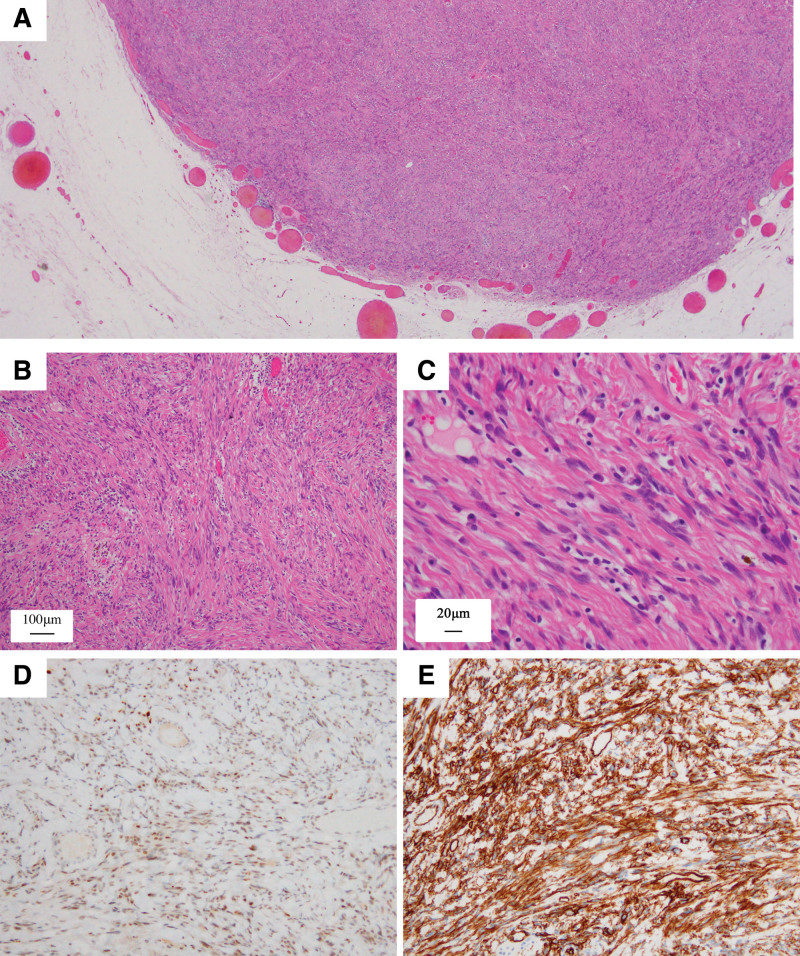
Microscopic findings (a) Well-circumscribed tumor with prominent peripheral vasculature, showing no infiltration into the surrounding adipose tissue (b) Spindle cells arranged haphazardly within a fibrous stroma (c) Uniform spindle cells with minimal pleomorphism and mitosis (d, e) Positive immunohistochemical staining for (d) signal transducer and activator of transcription 6 (strong and diffuse nuclear expression) and (e) cluster of differentiation 34; (hematoxylin and eosin stain; (a) × 20, (b) × 100, (c) × 400, (d) × 200 and (e) × 200).

The performed core-needle biopsy revealed a hyper-cellular spindle-cell tumor with a network of sinusoidal vessels but no cytological atypia, necrosis, or remarkable mitosis (<1 mitosis per 10 high-power field [HPF]). Grossly, the surgical specimen was composed of a well-circumscribed whitish firm mass, measuring 1.4 × 1.1 × 1.0 cm. The cut surface showed homogenously white and smooth appearance with multiple focal brownish/grayish areas, and no evidence of hemorrhage or necrosis. Microscopically, the mass showed a well-defined fibrous capsule, and no evidence of infiltration into the surrounding adipose tissue (Fig. 4a); displaying the same nuclear and mitotic morphology as shown in the core-needle biopsy. Spindle cells were arranged without pattern, with fibrillary collagen fibers, and alternating hypercellular and hypocellular areas (Fig. 4b and c). In the surgical specimen, tumor cells were positive for STAT6 and CD34 (Fig. 4d and e). Proliferative index of Ki67 was low. According to the morphological and immunohistochemical findings, the final diagnosis was SFT of the breast. IHC analysis was performed using a BenchMark GX automated staining instrument (Ventana Medical Systems, Inc.), and the different immunohistochemical markers applied are summarized in Table S2, http://links.lww.com/MD/I76.

## 4. Discussion

After Klemperer and Robin reported the first case of SFT in the pleura in 1931, extrapleural SFTs were infrequently reported at various body sites. Of the extrapleural SFTs, breast SFT remains the rarest, and histopathological and image diagnosis is both difficult and unsecured. In recent studies, NAB2-STAT6 or NFIX-STAT6 fusion gene was reported to be the driver mutation of SFT, which plays a key role in collagen production, vessel formation, and tumor proliferation^[3,4,6]^; however, the IHC staining of preoperative STAT6 staining for breast SFTs are seldom reported, possibly due to its rarity.

To our knowledge, 32 cases of breast SFT have been reported in previous case studies (Table S1, http://links.lww.com/MD/I209). Breast SFT seems to be more common in female (24 cases) than male (8 cases), and the morphological features did not significantly differ by sex; however, the IHC of actin and desmin tended to be more frequently positive in male patients (Table 1). Sex difference may have significant clinical importance in the management of this disease in clinical settings. Especially for female patients, it is difficult to distinguish breast SFTs from fibroadenoma, phyllodes tumor, and mammary hamartoma detected by ultrasound since breast SFT is often presented as an oval or lobulated well-defined hypoechoic lesion as shown in Table 1. Breast SFT rarely show calcification and homogeneous echo pattern unlike old fibroadenoma, but these do not provide definitive diagnosis. As seen in a case report of a female patient with breast SFT,^[7]^ the small lesion may be judged as not necessary for tissue sampling in ultrasound examination. In some cases,^[8,9]^ breast SFTs rapidly increased in size within a year, whether it was benign or malignant; however, in most cases, it gradually gets bigger over several years. Tissue sampling may be worth considering when well-circumscribed hypoechoic lesion showing hypervascularity exhibits a gradual growth in the breast during follow-up period.

**Table 1:** Comparison of morphological features and immunohistochemistry findings of breast SFT between male and female patients.

Features	Male (n = 9)	Female (n = 22)	*P* value
Age †	63.8 ± 12.0	61.0 ± 13.5	.60
**Ultrasound findings**			
Size (cm) †	2.6 ± 1.0	3.8 ± 2.8	.23
Shape			
Oval/round	4 (80)	5 (50)	
Lobulated	1 (20)	5 (50)	
NA	4	12	
Border			
Well-circumscribed	5 (100)	8 (80)	
Microlobulated	0 (0)	2 (20)	
NA	4	12	
Echotexture			
Hypoechoic	4 (80)	8 (89)	
Isoechoic to hyperechoic	1 (20)	1 (11)	
NA	4	13	
Internal echo			
Heterogeneous	3 (75)	7 (88)	
Homogeneous	1 (25)	1 (13)	
NA	5	14	
Vascularity			
Hypervascular	3 (100)	5 (83)	
Hypovascular	0 (0)	1 (17)	
NA	6	16	
**Immunohistochemistry**			
STAT6			
Positive	2 (67)	7 (100)	
Negative	1 (33)	0	
NA	6	15	
CD34			
Positive	8 (88)	22 (100)	
Negative	1 (11)	0 (100)	
NA	0	0	
Bcl-2			
Positive	3 (100)	11 (85)	
Negative	0 (0)	2 (15)	
NA	6	9	
CD99			
Positive	2 (100)	6 (100)	
Negative	0 (0)	0 (0)	
NA	7	16	
Actin			
Positive	3 (43)	3 (15)	
Negative	4 (57)	17 (85)	
NA	2	2	
Desmin			
Positive	4 (57)	0 (0)	
Negative	3 (43)	14 (100)	
NA	2	8	
S100			
Positive	0 (0)	0 (0)	
Negative	5 (100)	18 (100)	
NA	4	4	

− = negative, + = positive, Bcl-2 = B-cell lymphoma 2, CD = cluster of differentiation, f = focal, NA = not available, S100 = S100 protein, SFT = solitary fibrous tumor, STAT6 = signal transducer and activator of transcription 6.

† Data represent means ± standard deviation, compared using a Student *t* test.

In histology, SFT shows a typical pattern-less growth of the spindle cells, alternating with hypercellular and hypocellular areas, and needs to be distinguished from other spindle cell lesions, such as myofibroblastoma, fibroblastic spindle cell tumor, spindle cell lipoma, fibroma and pseudoangiomatous stromal proliferation. When breast SFT exhibited dedifferentiation or lipomatous change in morphological variant cases,^[10,11]^ malignant phyllodes tumor and liposarcoma should also be included in the differential diagnosis. In IHC analysis, a combination of CD34, Bcl-2, and CD99 positivity was traditionally used for the diagnosis of breast SFT; however, it was insufficient for the definitive diagnosis because approximately 10% of breast SFT was negative for CD34, while Bcl-2 and CD99 are more sensitive but less specific. In recent studies, a diffuse and intense STAT6 staining was reported to be the most effective diagnostic marker for SFT.^[12–14]^ Yoshida et al^[12]^ demonstrated that only 4 (2.5%) of 159 non-SFTs showed a weak nuclear expression of STAT6, and Koelsche et al^[13]^ reported that 5 (7.4%) of 68 dedifferentiated liposarcomas, 2 of 130 undifferentiated pleomorphic liposarcomas, and 1 (1.6%) of 63 cases of nodular fasciitis showed moderate to strong nuclear expression for STAT6. Demicco et al^[14]^ described a relatively higher rate (12%, 49 of 408 cases) of well-differentiated/dedifferentiated liposarcomas showing strong nuclear positivity for STAT6. However, the discrimination between the 2 will be easily achieved by histological evaluation when breast SFT is not accompanied by lipomatous change or dedifferentiation. In our case, myofibroblastoma should be traditionally considered in the differential diagnosis because not only CD 34, but also desmin and alpha-smooth muscle actin were positive. Diffuse and intense STAT6 positivity led to our final diagnosis of breast SFT with reference to the existing literature. The breast SFT showing both desmin and alpha-smooth muscle actin positivity is extremely rare, with only 4 previously reported cases.^[15]^

The traditional prognostic criteria of malignant SFTs was based on the clinicopathologic review of 223 cases of pleural SFTs published in 1989, and it included high cellularity, mitotic activity (more than 4 mitotic figures per 10 HPFs), pleomorphism, hemorrhage, and necrosis^[16]^; however, a multivariate risk stratification model for metastasis of non-meningeal SFTs established by Demicco et al in 2017 seems to be more widely prevalent.^[17]^ The risk score model included 4 predictors: age (≥55 vs <55), tumor size (≥15 vs 10 to < 15 vs 5 to < 10 vs <5 cm), mitotic count per 10 HPF (≥4 vs 1 to 3 vs 0), and tumor necrosis (≥10% vs <10%). According to the risk score, patients were divided into 3 groups: low-, intermediate-and high-risk groups. The 5-year disease free survival rate was validated as 100% for low-risk group, 90% for intermediate-risk group, and 27% for high-risk group. The calculated risk score in our case was within the low-risk range, however, careful follow-up was considered necessary, especially within the first 2 years after surgery, since most cases of local recurrence and distant metastasis occurred during that period.^[18,19]^ For breast SFT, only 1 case of local recurrence after excisional biopsy has been previously reported.^[20]^ Although breast SFT may have better prognosis as compared to other extrapleural SFTs, the lesion should be treated with complete surgical resection for the prevention of local recurrence. Therefore, to obtain a confident preoperative diagnosis of breast SFT at an early-stage is clinically important, and diffuse and strong nuclear expression of STAT6 is the most effective diagnostic method.

In conclusion, breast SFT is an exceedingly rare mesenchymal tumor, and male breast SFT has been reported only in 8 cases. In our case, the diffuse and strong nuclear immunostaining of STAT6 was particularly helpful for the preoperative diagnosis of SFT due to the positivity of desmin and alpha-smooth muscle actin. We could perform a complete surgical resection, which is the gold standard treatment for breast SFT. We believe that correct diagnosis at an early-stage utilizing STAT6 staining and careful follow-up will lead to a better prognosis and management of this disease.

This work was financially supported by the Okinaka Memorial Institute for Medical Research.

## Author contributions

**Conceptualization:** Shun Kawaguchi, Hidetaka Kawabata.

**Data curation:** Shun Kawaguchi, Keiichi Kinowaki, Hidetaka Kawabata.

**Formal analysis:** Shun Kawaguchi, Keiichi Kinowaki.

**Funding acquisition:** Hidetaka Kawabata.

**Methodology:** Shun Kawaguchi, Keiichi Kinowaki.

**Project administration:** Shun Kawaguchi.

**Resources:** Shun Kawaguchi.

**Supervision:** Shun Kawaguchi, Keiichi Kinowaki.

**Visualization:** Shun Kawaguchi.

**Writing – original draft:** Shun Kawaguchi.

**Writing – review & editing:** Shun Kawaguchi, Keiichi Kinowaki, Nobuko Tamura, Aya Nishikawa, Akio Shibata, Kiyo Tanaka, Yoko Kobayashi, Takuya Ogura, Junichiro Sato, Hidetaka Kawabata.

## Supplementary Material
