# Supplementary material for: Solitary fibrous tumor of male breast: A case report and literature review
Source: Medicine (Baltimore). 2022 Dec 16;101(50):e32199. doi: 10.1097/MD.0000000000032199 (PMC9771286; doi:10.1097/MD.0000000000032199)
Supplement: Supplementary file 2 [file medi-101-e32199-s002.pdf]

Supplementary Table 2 Summary of antibody reagents for immunohistochemical staining

Antibody	Clone	Dilution	Manufacturer
STAT 6	D-1	1:2000	Santa Cruz
CD34	790-2927	RTU	Roche
αSMA	M0851	1:1000	DAKO
desmin	IR606	1:2	DAKO
S100	Z0311	1:2000	DAKO
Ki67	790-4286	RTU	Roche

Abbreviations: STAT6, signal transducer and activator of transcription 6; CD, cluster of differentiation; S100, S100 protein; RTU, ready-to-use
