# Supplementary material for: Solitary fibrous tumor of male breast: A case report and literature review
Source: Medicine (Baltimore). 2022 Dec 16;101(50):e32199. doi: 10.1097/MD.0000000000032199 (PMC9771286; doi:10.1097/MD.0000000000032199)
Supplement: Supplementary file 3 [file medi-101-e32199-s003.pdf]

Supplementary Table 1 References

[1] Ben Ghashir NS, Balalaa NA, Anam W, Mohamed RM. Lipomatous (Fat-Forming) solitary fibrous tumor of the breast: a case report of an uncommon variant of a rare clinical entity. Case Rep Oncol 2022;15:455–461. https://doi.org/10.1159/000524364.

[2] Nitta T, Kimura K, Tominaga T, et al. Malignant solitary fibrous tumor of the breast. Breast J 2021;27:391–393. https://doi.org/10.1111/tbj.14175.

[3] Dubois C, Nika E, Hoffmann P, Delouche A, Michy T, Philippe AC. Solitary fibrous tumor of the breast: a rare entity. Breast J 2020;26:289–290. https://doi.org/10.1111/tbj.13537.

[4] Barco I, González C, Vallejo E, Pessarrodona A, Giménez N, García-Fernández A. Malignant solitary fibrous tumour of the breast mimicking a benign tumor. Clin Pathol 2019;12:2632010x19868462. https://doi.org/10.1177/2632010X19868462.

[5] Jung MJ, Alrahwan D, Dubrovsky E, Baek D, Ayala AG, Ro JY. Solitary fibrous tumor of breast with anaplastic areas (malignant solitary fibrous tumor): a case report with review of literature. J Breast Cancer 2019;22:326–235. https://doi.org/10.4048/jbc.2019.22.e30.

[6] Salemis NS. Solitary fibrous tumor of the breast: a case report and the review of the literature. Breast J 2018;24:78–81. https://doi.org/10.1111/tbj.12841.

[7] Song HS, Lee JY, Kim SY, et al. Solitary fibrous tumor of the male breast: a case report with imaging findings. Iran J Radiol 2018;(In Press). https://doi.org/10.5812/iranjradiol.65607.

[8] Brenes J, Moreno A, Merchán MJ, et al. Solitary fibrous tumor of the breast: a rare neoplasm. Breast J 2018;24:417–419. https://doi.org/10.1111/tbj.12925.

[9] Magro G, Spadola S, Motta F, et al. STAT6 expression in spindle cell lesions of the breast: an immunohistochemical study of 48 cases. Pathol Res Pract 2018;214:1544–1549. https://doi.org/10.1016/j.prp.2018.07.011.

[10] Magro G, Angelico G, Righi A, Benini S, Salvatorelli L, Palazzo J. Utility of STAT6 and 13q14 deletion in the classification of the benign spindle cell stromal tumors of the breast. HumPathol 2018;81:55–64. https://doi.org/10.1016/j.humpath.2018.06.015.

[11] Magro G, Angelico G, Leone G, Palazzo J. Solitary fibrous tumor of the breast: report of a case with emphasis on diagnostic role of STAT6 immunostaining. Pathol Res Pract 2016;212:463–467. https://doi.org/10.1016/j.prp.2015.12.013.

[12] Magro G, Bisceglia M, Michal M, Eusebi V. Spindle cell lipoma-like tumor, solitary fibrous tumor and myofibroblastoma of the breast: a clinico-pathological analysis of 13 cases in favor of a unifying histogenetic concept. Virchows Archiv 2002;440:249–260. https://doi.org/10.1007/s00428-001-0572-y.
[13] Magro G, Sidoni A, Bisceglia M. Solitary fibrous tumour of the breast: distinction from myofibroblastoma. Histopathology 2000;37:189–191. https://doi.org/10.1046/j.1365-2559.2000.00985-3.x.
[14] Park BN, Woo OH, Kim C, Cho KR, Seo BK. Recurrent solitary fibrous tumor of the breast: magnetic resonance imaging and pathologic findings. Breast J 2018;24:1064–1065. https://doi.org/10.1111/tbj.13131.
[15] Tsai SY, , Hsu CY, Chou YH, et al. Solitary fibrous tumor of the breast: A case report and review of the literature. J Clin Ultrasound 2017;45:350–354. https://doi.org/10.1002/jcu.22415.
[16] Riola-Parada C, Jiménez-Ballvé A, Serrano-Palacio A, Cabrera-Martín MN, Brenes-Sánchez JM, Carreras-Delgado JL. Solitary fibrous breast tumour: Contribution of (18)F-FDG PET/CT. Rev Esp Med Nucl Imagen Mol 2017;36:61–62. https://doi.org/ 10.1016/j.remn.2016.01.002.
[17] Rhee SJ, Ryu JK, Han SA, Won KY. Solitary fibrous tumor of the breast: a case report and review of the literature. J Med Ultrason (2001) 2016;43:125–128. https://doi.org/10.1007/s10396-015-0674-9.
[18] Han Y, Zhang Q, Yu X, et al. Immunohistochemical detection of STAT6, CD34, CD99 and BCL-2 for diagnosing solitary fibrous tumors/hemangiopericytomas. Int J Clin Exp Pathol 2015;8:13166–133175.
[19] Yang LH, Dai SD, Li QC, et al. Malignant solitary fibrous tumor of breast: a rare case report. Int J Clin Exp Pathol 2014;7:4461–4466.
[20] Wignall OJ, Moskovic EC, Thway K, Thomas JM. Solitary fibrous tumors of the soft tissues: review of the imaging and clinical features with histopathologic correlation. AJR Am J Roentgenol 2010;195:W55–W62. https://doi.org/10.2214/AJR.09.3379.
[21] Rovera F, Imbriglio G, Limonta G, et al. Solitary fibrous tumor of the male breast: a case report and review of the literature. World J Surg Oncol 2008;6:16. https://doi.org/10.1186/1477-7819-6-16.
[22] Meguerditchian AN, Malik DA, Hicks DG, Kulkarni S. Solitary fibrous tumor of the breast and mammary myofibroblastoma: the same lesion? Breast J 2008;14:287–292. https://doi.org/10.1111/j.1524-4741.2008.00588.x.

[23] Falconieri G, Lamovec J, Mirra M, Pizzolitto S. Solitary fibrous tumor of the mammary gland: a potential pitfall in breast pathology. Ann Diagn Pathol 2004;8:121–125. https://doi.org/10.1016/j.anndiagpath.2004.03.002.

[24] Bombonati A, Parra JS, Schwartz GF, Palazzo JP. Solitary fibrous tumor of the breast. Breast J 2003;9:251–251. https://doi.org/10.1046/j.1524-4741.2003.09315.x.

[25] Salomão DR, Crotty TB, Nascimento AG. Myofibroblastoma and solitary fibrous tumour of the breast: histopathologic and immunohistochemical studies. Breast 2001;10:49–54. https://doi.org/10.1054/brst.2000.0188.

[26] Khalifa MA, Montgomery EA, Azumi N, et al. Solitary fibrous tumors: a series of lesions, some in unusual sites. South Med J 1997;90:793–799. https://doi.org/10.1097/00007611-199708000-00005.

[27] Damiani S, Miettinen M, Peterse JL, Eusebi V. Solitary fibrous tumour (myofibroblastoma) of the breast. Virchows Arch 1994;425:89–92. https://doi.org/10.1007/BF00193955.
